# Genetic Variants in *CASP3*, *BMP5*, and *IRS2* Genes May Influence Survival in Prostate Cancer Patients Receiving Androgen-Deprivation Therapy

**DOI:** 10.1371/journal.pone.0041219

**Published:** 2012-07-23

**Authors:** Shu-Pin Huang, Bo-Ying Bao, Tzyh-Chyuan Hour, Chao-Yuan Huang, Chia-Cheng Yu, Chia-Chu Liu, Yung-Chin Lee, Chun-Nung Huang, Jiunn-Bey Pao, Chun-Hsiung Huang

**Affiliations:** 1 Department of Urology, Kaohsiung Medical University Hospital, Kaohsiung, Taiwan; 2 Department of Urology, Faculty of Medicine, College of Medicine, Kaohsiung Medical University, Kaohsiung, Taiwan; 3 Cancer Center, Kaohsiung Medical University Hospital, Kaohsiung, Taiwan; 4 Department of Pharmacy, China Medical University, Taichung, Taiwan; 5 Sex Hormone Research Center, China Medical University Hospital, Taichung, Taiwan; 6 Institute of Biochemistry, Kaohsiung Medical University, Kaohsiung, Taiwan; 7 Department of Urology, National Taiwan University Hospital, Taipei, Taiwan; 8 Division of Urology, Department of Surgery, Kaohsiung Veterans General Hospital, Kaohsiung, Taiwan; 9 Department of Pharmacy, Yangming Branch, Taipei City Hospital, Taipei, Taiwan; Tor Vergata University of Rome, Italy

## Abstract

Several genome-wide association studies (GWAS) have been conducted to identify the common single nucleotide polymorphisms (SNPs) that influence the risk of prostate cancer. It was hypothesized that some prostate cancer-associated SNPs might relate to the clinical outcomes in patients treated for prostate cancer using androgen-deprivation therapy (ADT). A cohort of 601 patients who have received ADT for prostate cancer was genotyped for 29 SNPs that have been associated with prostate cancer in Cancer Genetic Markers of Susceptibility GWAS, and within the genes that have been implicated in cancer. Prognostic significance of these SNPs on the disease progression, prostate cancer-specific mortality (PCSM) and all-cause mortality (ACM) after ADT were assessed by Kaplan-Meier analysis and Cox regression model. Three SNPs, namely *CASP3* rs4862396, *BMP5* rs3734444 and *IRS2* rs7986346, were found to be closely associated with the ACM (*P*≤0.042), and *BMP5* rs3734444 and *IRS2* rs7986346 were also noted to be significantly related to the PCSM (*P*≤0.032) after adjusting for the known clinicopathologic predictors. Moreover, patients carrying a greater number of unfavorable genotypes at the loci of interest had a shorter time to ACM and PCSM during ADT (*P* for trend <0.001). Our results suggest that *CASP3* rs4862396, *BMP5* rs3734444 and *IRS2* rs7986346 may affect the survival in patients after ADT for prostate cancer, and the analysis of these SNPs can help identify patients at higher risk of poor outcome.

## Introduction

Prostate cancer, the most frequently diagnosed cancer, is the second leading cause of cancer-related deaths among men in Western countries [1], [2]. Since it was first introduced more than 70 years ago, an androgen-deprivation therapy (ADT) has been the mainstay of treatment for advanced prostate cancer based on that the androgen signaling has been considered the main oncogenic driver in prostate carcinogenesis. The initial response rate of prostate cancer to ADT has been reported to be up to 80%, however, the disease in most of treated patients have progressed toward castration-resistant prostate cancer (CRPC). Unfortunately, CRPC is still incurable and the median survival for patients with CRPC is only 1–2 years. Nevertheless, poor understanding of the molecular mechanisms underlying CRPC might be the constraint on development of efficient therapy.

Genetic biomarkers have been demonstrated to have potential for enabling application of more effective personalized diagnosis, prognosis and treatment in clinics. Recently, millions of single nucleotide polymorphisms (SNPs) have been identified to be associated with the risk of prostate cancer in several genome-wide association studies, such as the Cancer Genetic Markers of Susceptibility (CGEMS) study [3]–[14]. Nonetheless, the prognostic value of the prostate cancer-associated variants has not been well documented.

The purpose of the current study was to investigate the prognostic significance of 29 SNPs that were associated with genes implicated in cancer progression and had low *P* values (*P*<0.01) in CGEMS (Table S1) for disease progression, prostate cancer-specific mortality (PCSM) and all-cause mortality (ACM) in a cohort of 601 patients treated with ADT for prostate cancer.

## Methods

### Patient recruitment and data collection

Patients with diagnosed and pathologically confirmed prostate cancer were actively recruited from three medical centers in Taiwan: Kaohsiung Medical University Hospital and Kaohsiung Veterans General Hospital in southern Taiwan, and National Taiwan University Hospital in northern Taiwan. Written informed consent was obtained from each participant and permission to conduct this study was provided by the Institutional Review Board of the three hospitals. Collection of the clinical data and patient characteristics described as previous study [15]–[19] are reported in Method S1.

### Genotyping

Genomic DNA was extracted from the peripheral blood of each patient and genotyping was performed as described previously [20] using Sequenom iPLEX matrix-assisted laser desorption/ionization-time of flight (MALDI-TOF) mass spectrometry technology at the National Center for Genome Medicine, Academia Sinica, Taiwan. Briefly, primers for locus-specific polymerase chain reaction (PCR) and allele-specific extension were designed by MassARRAY AssayDesign 3.0 software (Sequenom, San Diego, CA, USA). For primer sequences, see Table S2. The sample DNAs were amplified by primers flanking the targeted sequence, followed by dephosphorylation and allele-specific primer extension. The extension products were purified, loaded into a 384-format SpectroChip, and subjected to MALDI-TOF mass spectrometry. The resulting data were analyzed by the Sequenom MassARRAY TYPER software (Sequenom, San Diego, CA, USA). The average genotype call rate for these SNPs was 99.3% and the concordance rate was 99.8% among 55 blind duplicated quality control samples. Each of the SNPs was in Hardy-Weinberg equilibrium (*P*>0.01).

### Real-time reverse transcription-PCR (real-time RT-PCR)

The human prostate cell lines, namely LNCaP, DU 145 and PC-3, were purchased from Bioresource Collection and Research Center (Hsinchu, Taiwan) and maintained in RPMI 1640 with 10% fetal bovine serum, 100 U/ml penicillin and 100 U/ml streptomycin (Invitrogen, Carlsbad, CA, USA) at 37°C in a humidified 5% CO_2_-95% air atmosphere. Total RNA was extracted from LNCaP, DU 145 and PC-3 cells using Trizol (Invitrogen, Carlsbad, CA, USA). Reverse transcription with the High Capacity cDNA Reverse Transcription Kit (ABI, Foster city, CA, USA) and PCR amplifications with Smart Quant Green Master Mix (Protech, Taipei, Taiwan) were carried out on an MJ mini and MiniOpticon real-time PCR detection system (Bio-Rad, Hercules, CA, USA). PCR was performed as the following sequence: initial HotStart activation at 95°C for 15 min, and 40 cycles of denaturation at 95°C for 15 s, annealing and extension at 60°C for 1 min. Primer sequences were: *caspase 3* (*CASP3*), sense 5′- GACATACTCCTTCCATCAA-3′ and antisense 5′- ATTCATAGCACAGCATCA-3′; *bone morphogenetic protein 5* (*BMP5*), sense 5′- CCGTCTTCTGCTACATCA-3′ and antisense 5′- ACAACATCCTCACCGATT-3′; *insulin receptor substrate 2* (*IRS2*), sense 5′- CAGTGTATTGACGCATAT-3′ and antisense 5′- AGCATATTATCATCTGTGTA-3′; *Glyceraldehyde-3-phosphate dehydrogenase* (*GAPDH*), sense 5′-TCACCACCATGGAGAAGGC-3′ and antisense 5′-GCTAAGCAGTTGGTGGTGCA-3′. Quantification of each sample relative to the LNCaP sample was calculated using 2^−ΔΔCT^ method [21]. The expected sizes and the absence of non-specific amplification products were confirmed by agarose gel electrophoresis and melting curve analysis.

### Statistical analysis

Patient's clinicopathologic characteristics were summarized as number and percentage of patients, median, or interquartile range (IQR) of values. The continuous factors were dichotomized at the median value within the cohort, with the exception of prostate-specific antigen (PSA) nadir that was dichotomized at 0.2 ng/mL because of its correlation with disease progression and PCSM [22], [23]. The heterozygous and rare homozygous genotypes were collapsed in the analysis if the frequency of the rare homozygote was too low (<2%), or if the homozygous and heterozygous genotypes had the same direction of effect. The associations of 29 individual SNPs and clinicopathologic characteristics with time to progression, PCSM and ACM were assessed using the Kaplan-Meier analysis with log-rank test. Multivariate analyses to determine the interdependency of genotypes and other known prognostic factors, such as age at diagnosis, clinical stage, Gleason score, PSA at ADT initiation, PSA nadir and time to PSA nadir, were carried out using Cox proportional hazards regression model. As the 29 SNPs were tested, the false-discovery rates (*q* values) were calculated to determine the degree to which tests for association were prone to false-positives [24]. *q* values were estimated using R *q* value package (http://genomics.princeton.edu/storeylab/qvalue/) on the observed distribution of *P* values from the log-rank test for 29 SNPs. Statistical Package for the Social Sciences software version 16.0.1 (SPSS Inc., Chicago, IL) was used for other statistical analyses. A two-sided *P* value of ≤0.05 was considered statistically significant.

## Results

The study population (*N* = 601) is derived from a previously described cohort of prostate cancer patients treated with ADT and its clinicopathologic characteristics are presented in Table S3. Clinical outcomes following ADT were measured by time to disease progression, PCSM and ACM. The clinical stage at diagnosis, PSA nadir and time to PSA nadir during ADT were significantly associated with all three clinical outcomes after ADT (*P*≤0.019). While age at diagnosis was only associated with ACM (*P* = 0.008), Gleason score at diagnosis and PSA level at ADT initiation were both associated with time to PCSM and ACM (*P*<0.001), but not with time to progression.

A total of 29 SNPs that were associated with genes implicated in cancer and had low *P* values (*P*<0.01) in CGEMS, were selected. Their associations with disease progression, PCSM and ACM after ADT were summarized in Table S1. Our primary log-rank tests revealed that *BMP5* rs3734444, *NCOR2* rs10846667, *IRS2* rs7986346 and *MAP2K6* rs1972933 significantly related to disease progression (nominal *P*≤0.043) with a false-discovery rate (*q* value) of 0.085 (Table 1). To evaluate the prognostic value of these SNPs beyond the currently used clinical factors, a multivariate Cox proportional hazards analysis adjusting for age, clinical stage, Gleason score at diagnosis, PSA nadir, time to PSA nadir and PSA at ADT initiation was performed. After adjusting for these predictors, no statistical association was observed between any SNPs and disease progression (*P*≥0.084, Table 1).

**Table 1:** Genotyping frequencies and the association of genotype with disease progression during ADT.

Gene SNP	Genotype	No. of Patients	No. of Events	Median (months)	*P* *	*q*	HR (95% CI)	*P* †
*BMP5* rs3734444	AA+AG	572	394	23	0.022	0.085	1.00	
	GG	21	17	15			1.56 (0.94–2.60)	0.087
*NCOR2* rs10846667	CC	395	266	24	0.011	0.085	1.00	
	CT+TT	192	142	18			1.16 (0.93–1.43)	0.184
*IRS2* rs7986346	TT+TG	512	355	21	0.040	0.085	1.00	
	GG	71	48	32			0.76 (0.56–1.04)	0.084
*MAP2K6* rs1972933	GG	208	141	25	0.043	0.085	1.00	
	GT+TT	387	272	21			1.19 (0.97–1.48)	0.102

Abbreviations: ADT, androgen-deprivation therapy; HR, hazard ratio; 95% CI, 95% confidence interval; PSA, prostate-specific antigen.

* *P* values were calculated using the log-rank test.

† HRs were adjusted for age, clinical stage, Gleason score, PSA at ADT initiation, PSA nadir, and time to PSA nadir.

**Figure 1:**
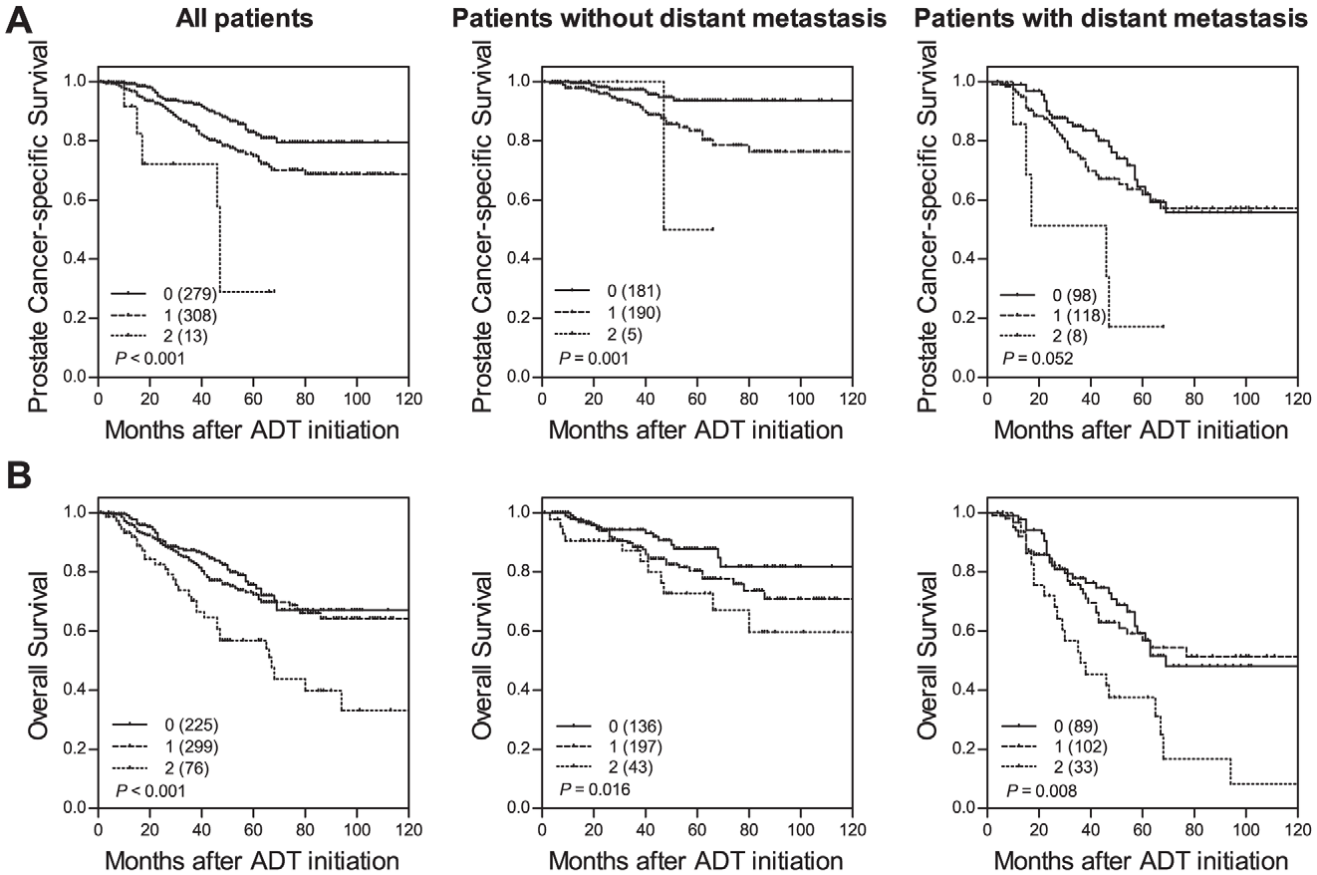
The influence of the genetic loci of interest on prostate cancer prognosis. Kaplan-Meier curves of (A) time to PCSM during ADT for patients with 0, 1, or 2 unfavorable genotypes at the 2 genetic loci of interest, and (B) time to ACM during ADT for patients with 0, 1, or >1 unfavorable genotypes at the 3 genetic loci of interest; measured in all patients (left), in patients without distant metastasis (middle), or in patients with distant metastases (right). Numbers in parentheses indicate the number of patients.

*BMP5* rs373444, *RXRA* rs3118536, *IRS2* rs7986346 and *ERG* rs2836370 were associated with PCSM with nominal *P*≤0.043 and *q*≤0.209 (Table 2). Six SNPs, namely *CASP3* rs4862396, *BMP5* rs373444, *RXRA* rs3118536, *BMPR1A* rs11597689, *IRS2* rs7986346 and *ERG* rs2836370, also exhibited significant influence on ACM (nominal *P*≤0.037), and all had a *q* value of ≤0.179 (Table 3). After adjusting for clinical predictors, *BMP5* rs3734444 and *IRS2* rs7986346 remained significant association with both PCSM (adjusted *P*≤0.032, Table 2) and ACM (adjusted *P*≤0.034, Table 3). A strong gene-dosage effect on PCSM during ADT was noted when *BMP5* rs3734444 and *IRS2* rs7986346 were analyzed in combination (log-rank *P*<0.001, Table 2 and Figure 1A left), and the hazard ratios (HRs) increased as the number of unfavorable genotypes increased. In addition, the association of *CASP3* rs4862396 with time to ACM also remained significant (adjusted *P* = 0.042, Table 3) when other risk factors were included in the multivariate analysis. Estimated time to ACM decreased progressively for patients with increasing number of unfavorable genotypes for *BMP5* rs3734444, *IRS2* rs7986346 and *CASP3* rs4862396 (Table 3 and Figure 1B left). The risk of ACM increased in patients with 1 unfavorable genotype (HR 1.43, 95% confidence interval [CI] 0.97–2.11) and significantly increased for patients with >1 unfavorable genotypes (HR 2.48, 95% CI 1.54–3.98) when compared to patients without unfavorable genotypes (*P* for trend <0.001, Table 3).

**Table 2:** Genotyping frequencies and the association of genotype with PCSM during ADT.

Gene SNP	Genotype	No. of Patients	No. of Events	Estimated Mean (months)	*P* *	*q*	HR (95% CI)	*P* †
***BMP5*** ** rs3734444**	AA+AG	575	93	135	0.002	0.039	1.00	
	GG	21	8	96			**2.28 (1.08–4.82)**	**0.032**
*RXRA* rs3118536	CC	392	75	130	0.043	0.209	1.00	
	CA+AA	204	25	148			0.74 (0.47–1.16)	0.189
***IRS2*** ** rs7986346**	TT	272	35	147	0.007	0.068	1.00	
	TG+GG	313	65	122			**1.90 (1.25–2.90)**	**0.003**
*ERG* rs2836370	TT+TC	506	92	128	0.035	0.209	1.00	
	CC	85	7	157			0.50 (0.23–1.08)	0.079
No. of Unfavorable Genotypes Present‡								
0		279	34	143	<0.001		1.00	
**1**		308	61	132			**1.94 (1.26–2.99)**	**0.003**
**2**		13	6	44			**3.90 (1.60–9.52)**	**0.003**
							*P*-trend	**<0.001**

Abbreviations: ADT, androgen-deprivation therapy; HR, hazard ratio; 95% CI, 95% confidence interval; PSA, prostate-specific antigen.

* *P* values were calculated using the log-rank test.

† HRs were adjusted for age, clinical stage, Gleason score, PSA at ADT initiation, PSA nadir, and time to PSA nadir.

‡ Unfavorable genotypes refer to GG in BMP5 rs3734444 and TG+GG in IRS2 rs7986346.

*P*≤0.05 are in boldface.

**Table 3:** Genotyping frequencies and the association of genotype with ACM during ADT.

Gene SNP	Genotype	No. of Patients	No. of Events	Estimated Mean (months)	*P* *	*q*	HR (95% CI)	*P* †
***CASP3*** ** rs4862396**	TT	475	105	127	0.037	0.179	1.00	
	TC+CC	123	39	102			**1.49 (1.01–2.19)**	**0.042**
***BMP5*** ** rs3734444**	AA+AG	575	136	120	0.014	0.138	1.00	
	GG	21	9	80			**2.13 (1.06–4.28)**	**0.034**
*RXRA* rs3118536	CC	392	108	114	0.016	0.138	1.00	
	CA+AA	204	36	134			0.68 (0.47–1.01)	0.054
*BMPR1A* rs11597689	GG	523	134	116	0.028	0.162	1.00	
	GA+AA	76	11	146			0.64 (0.34–1.21)	0.172
***IRS2*** ** rs7986346**	TT	272	54	133	0.017	0.138	1.00	
	TG+GG	313	87	109			**1.63 (1.15–2.32)**	**0.006**
*ERG* rs2836370	TT+TC	506	132	123	0.019	0.138	1.00	
	CC	85	11	149			0.55 (0.30–1.03)	0.061
No. of Unfavorable Genotypes Present‡								
0		225	45	128	<0.001		1.00	
1		299	68	126			1.43 (0.97–2.11)	0.069
**>1**		76	32	84			**2.48 (1.54–3.98)**	**<0.001**
							*P*-trend	**<0.001**

Abbreviations: ADT, androgen-deprivation therapy; HR, hazard ratio; 95% CI, 95% confidence interval; PSA, prostate-specific antigen.

* *P* values were calculated using the log-rank test.

† HRs were adjusted for age, clinical stage, Gleason score, PSA at ADT initiation, PSA nadir, and time to PSA nadir.

‡ Unfavorable genotypes refer to TC+CC in CASP3 rs4862396, GG in BMP5 rs3734444, and TG+GG in IRS2 rs7986346.

*P*≤0.05 are in boldface.

Since patients with distant metastasis are considered high risk, patients were further stratified based on their metastatic status at the initiation of ADT for evaluating the clinical relevance of these SNPs. The combined genotypes still had effects on PCSM and ACM in patients with or without distant metastasis, respectively (*P*≤0.052, Figure 1 middle and right). These results support that these SNPs could be the independent survival predictors following ADT, along with the current clinicopathologic prognostic markers. Integration of these SNPs with the known predictors may improve risk stratification and help making treatment decisions.

**Figure 2:**
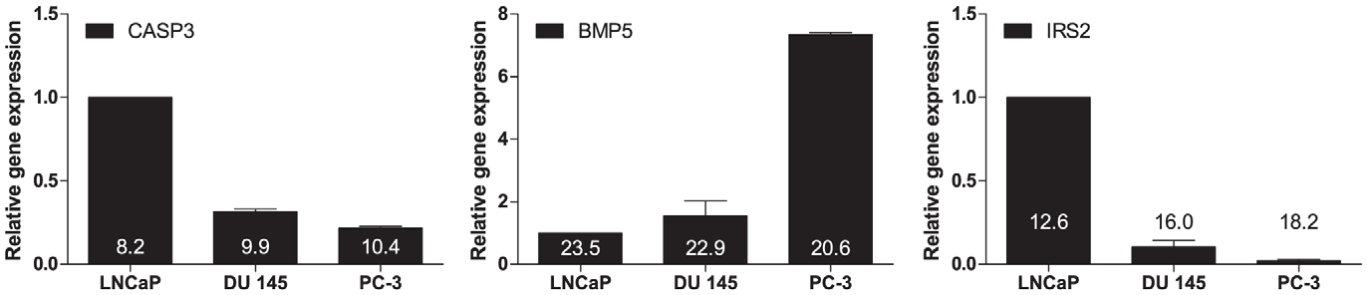
The mRNA expressions of endogenous *CASP3*, *BMP5* and *IRS2* in human prostate cancer cell lines. Total RNAs were prepared from LNCaP, DU 145 and PC-3 cells, and gene expressions were analyzed using real-time RT-PCR. Relative gene expression represents the fold changes in gene expression relative to LNCaP cells set at 1.0. Numbers on the bars represent the difference in threshold cycles between genes of interest and internal control gene. Data are expressed as the mean ± SE of three independent experiments.

To gain an initial indication of these candidate genes in prostate cancer, the expression levels of *CASP3*, *BMP5* and *IRS2* in three most commonly used human prostate cancer cell lines, namely LNCaP, DU 145 and PC-3, were examined using real-time RT-PCR (Figure 2). It has been well established that LNCaP cells express androgen receptor (AR) and are androgen-sensitive with markedly less invasive potential [25]. DU 145 and PC-3 cells, on the other hand, do not express AR and are androgen-insensitive with highly aggressive behavior [26]. mRNA for all three genes were able to be detected in all prostate cancer cell lines. *CASP3* is one of the main executors of apoptosis. The expression of endogenous *CASP3* transcripts was inversely correlated to the aggressiveness in human prostate cancer cell lines, showing that it was higher in LNCaP than in DU 145 and PC-3. Elevated expression of BMPs has been implicated in prostate cancer, particularly in the disease-specific bone-metastasis [27]. A positive correlation was observed between the metastatic potential of these prostate cancer cell lines and *BMP5* expression. Although *IRS2* has been positively associated with aggressive tumor behavior [28], it has also been identified as a primary target gene of AR [29]. The endogenous *IRS2* expressed higher levels in AR-positive LNCaP cells than in AR-negative DU 145 and PC-3 cells. All of these independent lines of evidence support the biological plausibility of our suggestion that the risk variants of prostate cancer may also affect the survival in patients treated with ADT for prostate cancer.

## Discussion

This study aimed to investigate the prognostic effect of 29 prostate cancer-associated SNPs selected from CGEMS in patients receiving ADT for prostate cancer. Our results revealed that *CASP3* rs4862396, *BMP5* rs3734444 and *IRS2* rs7986346 were significantly associated with ACM, and *BMP5* rs3734444 and *IRS2* rs7986346 were also significantly related to PCSM. In addition, multivariate analysis showed that these SNPs were independent prognostic factors after adjusting for the known clinicopathologic predictors, including age, clinical stage, Gleason score at diagnosis, PSA nadir, time to PSA nadir and PSA at ADT initiation. It suggested that *CASP3* rs4862396, *BMP5* rs3734444 and *IRS2* rs7986346 may be useful markers for predicting the survival in patients receiving ADT for prostate cancer. To the best of our knowledge, this is the first study to determine whether the genetic variants in *CASP3*, *BMP5* and *IRS* genes have prognostic effects on the clinical outcomes of patients with prostate cancer.

*CASP3* encodes a member of the caspase family, which is responsible for apoptosis execution. Once CASP3 is proteolytically activated by the initiator caspases during death receptor- or DNA-damage-induced apoptosis, it cleaves a large set of substrates and results in the disassembly of the cell. Although the molecular mechanisms by which prostate cancer cells survive under ADT are unknown, dysregulation of survival/apoptosis proteins has been suspected to render cancer cells less prone to cell death induced by androgen deprivation [30]. rs4862396 resides 5-kb downstream of the *CASP3* gene. Although the function predicted for rs4862396 has not been known, several untyped *CASP3* SNPs are tightly linked (*r* ^2^>0.9) with rs4862396. According to the prediction of SNP Function Portal [31], many of the linked SNPs are able to alter binding sites for transcription factors (i.e. rs7675251 in hepatocyte nuclear factor 1 binding site), target sites for microRNAs (i.e. rs8549 in the target site of hsa-miR-196a), and amino acid sequence (i.e. rs1049210 leading to E189D). Therefore, further fine mapping of *CASP3* gene might help the identification of the strongest markers and improve our understanding of the contribution of *CASP3* rs4862396 to prostate cancer progression.

*BMP5* encodes a member of the BMP family which belongs to the transforming growth factor-β superfamily. Since BMPs are potent regulators for bone homeostasis and prostate cancer is most likely to metastasize to the bone, there is an increasing interest to investigate the roles of BMPs in prostate cancer bone metastasis. The expression levels of several BMPs in prostate cancer have been linked with the acquisition of osteogenic characteristics and the tumor progression to bone [32]–[34]. Furthermore, amplifications of the *BMP5* gene loci appeared to be more prevalent in tumors with a higher Gleason score and occurred in 50% of prostate tumors, which might account for the abnormal gene expression patterns of BMP5 in prostate cancer [27]. rs3734444 is located in the exon 1 of the *BMP5* gene. Although rs3734444 is a synonymous SNP and does not affect amino acid sequence, it is predicted to alter binding sites for the transcription factor CP2 and myocyte enhancer factor 2A. In addition, rs3734444 is also predicted to locate at the binding sites for exonic splicing silencer and enhancer, which play important roles in constitutive and alternative splicing. Therefore, it is possible that rs3734444 might directly or indirectly, through other linked SNPs, influence BMP5 expression, interaction between prostate cancer cells and the bone microenvironment, and ultimately progression to bone metastasis.

*IRS2* encodes a member of a family of intracellular signaling adaptor proteins that coordinate numerous biologically key extracellular signals within the cell, including insulin, insulin-like growth factor 1 (IGF1), hormones, cytokines and integrins. Alterations in IRS expression have been linked to not only metabolic diseases but also many types of cancer [35]–[37]. Upon ligand-binding, insulin receptor and IGF1 receptor are autophosphorylated and present docking sites for IRSs, which are phosphorylated by the receptor tyrosine kinase. The phospho-IRSs recruit and activate many downstream pathways: the two best studied being the phosphatidylinositol 3-kinase (PI3K)/phosphatase and tensin homolog (PTEN) and the extracellular signal-regulated kinase pathways. Deletion of *IRS2* has been found to suppress prostate cancer cell growth, proliferation and invasion in *PTEN* ^+/−^ mice by decreasing MYC expression and DNA synthesis [28]. While ADT controls the prostate cancer growth, it is also associated with a pattern of metabolic alterations, such as hyperinsulinaemia [38]. These high circulating levels of serum insulin has been shown to act directly on CRPC cells and to enhance *de novo* androgen synthesis through upregulation of IRS2 and several downstream steroidogenic enzymes [39]. rs7986346 located at 15-kb upstream of the *IRS2* gene was predicted to alter CCAAT/enhancer binding protein γ by SNP Function Portal. Therefore, rs7986346 might regulate IRS2 expression and affect prostate cancer progression to castrate resistance.

In conclusion, this study lights up several pathways to influence the survival after ADT, such as *CASP3* in apoptosis, *BMP5* in bone homeostasis and *IRS2* in steroidogenesis, and the cell survival. *CASP3* rs4862396, *BMP5* rs3734444 and *IRS2* rs7986346 were found to be the independent prognostic markers for patients receiving ADT for prostate cancer. It suggest that, in addition to the currently used clinicopathologic predictors, test of the three SNPs might help identify the patient subgroups at higher risk for poor outcome, thus assisting in tailoring individual therapeutic interventions for advanced prostate cancer. However, this study is still limited by sample size in subset analyses and multiple comparisons. In addition, our homogeneous Chinese Han population may make our findings less generalizable to other ethnic groups. Further functional analyses and large independent studies in other ethnic populations are necessary to validate the relevance of the observed associations to the efficacy of ADT for prostate cancer.

## Supporting Information

Method S1 **Patient recruitment and data collection.**
(DOC)

Table S1 **Genotyped SNPs and the** ***P*** **values of their association with time to progression, PCSM, ACM during ADT.**
(DOC)

Table S2 **Oligonucleotides used for genotyping analysis.**
(DOC)

Table S3 **Distribution of clinicopathologic characteristics and their associations with disease progression, PCSM, and ACM in prostate cancer patients receiving ADT.**
(DOC)
