# Supplementary material for: Genetic Variants in CASP3, BMP5, and IRS2 Genes May Influence Survival in Prostate Cancer Patients Receiving Androgen-Deprivation Therapy
Source: PLoS One. 2012 Jul 23;7(7):e41219. doi: 10.1371/journal.pone.0041219 (PMC3402522; doi:10.1371/journal.pone.0041219)
Supplement: Method S1 — Patient recruitment and data collection. (DOC) [file pone.0041219.s001.doc]

**Method S1.** Patient recruitment and data collection

The study population was extended from our hospital-based prostate cancer case-control study that has been described previously . Briefly, patients with diagnosed and pathologically confirmed prostate cancer were actively recruited from 3 medical centers in Taiwan: Kaohsiung Medical University Hospital, Kaohsiung Veterans General Hospital and National Taiwan University Hospital. Patients who had been treated using ADT for prostate cancer (orchiectomy or LHRH agonist with or without antiandrogen), including those with disease recurrence after local treatments, were identified and followed up prospectively to evaluate genetic variants as prognostic predictors of clinical outcomes during ADT. Patients were excluded if the clinicopathologic information or follow-up period were insufficient, leaving 601 patients in this cohort. This study was approved by the Institutional Review Board of the 3 hospitals, and written informed consent was obtained from each participant.

Data were collected on patients with disease baseline and clinicopathologic characteristics, as well as 3 treatment outcomes: time to progression, PCSM and ACM. The PSA nadir was defined as the lowest PSA value achieved during ADT treatment . Time to PSA nadir was defined as the duration of time it took for the PSA value to reach nadir after ADT initiation . Disease progression was defined as a serial rise in PSA, at least 2 rises in PSA (> 1 week apart), greater than the PSA nadir . Initiation of secondary hormone treatment for rising PSA was also considered as a progression event. Time to progression was defined as the duration of time it took to have a progression event once ADT was started. In general, patients are followed every month with PSA tests at 3-monthly intervals. The cause of death was obtained by matching patients’ personal identification number with the official cause of death registry provided by the Department of Health, Executive Yuan, Taiwan. Overall, 145 deaths were identified and 101 of them died from prostate cancer.

**References**

1. Huang SP, Chou YH, Wayne Chang WS, Wu MT, Chen YY, et al. (2004) Association between vitamin D receptor polymorphisms and prostate cancer risk in a Taiwanese population. Cancer Lett 207: 69-77.

2. Huang SP, Huang CY, Wang JS, Liu CC, Pu YS, et al. (2007) Prognostic significance of p53 and X-ray repair cross-complementing group 1 polymorphisms on prostate-specific antigen recurrence in prostate cancer post radical prostatectomy. Clin Cancer Res 13: 6632-6638.

3. Huang SP, Huang CY, Wu WJ, Pu YS, Chen J, et al. (2006) Association of vitamin D receptor FokI polymorphism with prostate cancer risk, clinicopathological features and recurrence of prostate specific antigen after radical prostatectomy. Int J Cancer 119: 1902-1907.

4. Huang SP, Huang LC, Ting WC, Chen LM, Chang TY, et al. (2009) Prognostic significance of prostate cancer susceptibility variants on prostate-specific antigen recurrence after radical prostatectomy. Cancer Epidemiol Biomarkers Prev 18: 3068-3074.

5. Huang SP, Ting WC, Chen LM, Huang LC, Liu CC, et al. (2010) Association analysis of Wnt pathway genes on prostate-specific antigen recurrence after radical prostatectomy. Ann Surg Oncol 17: 312-322.

6. Huang SP, Wu WJ, Chang WS, Wu MT, Chen YY, et al. (2004) p53 Codon 72 and p21 codon 31 polymorphisms in prostate cancer. Cancer Epidemiol Biomarkers Prev 13: 2217-2224.

7. Stewart AJ, Scher HI, Chen MH, McLeod DG, Carroll PR, et al. (2005) Prostate-specific antigen nadir and cancer-specific mortality following hormonal therapy for prostate-specific antigen failure. J Clin Oncol 23: 6556-6560.

8. Kwak C, Jeong SJ, Park MS, Lee E, Lee SE (2002) Prognostic significance of the nadir prostate specific antigen level after hormone therapy for prostate cancer. J Urol 168: 995-1000.

9. Choueiri TK, Xie W, D'Amico AV, Ross RW, Hu JC, et al. (2009) Time to prostate-specific antigen nadir independently predicts overall survival in patients who have metastatic hormone-sensitive prostate cancer treated with androgen-deprivation therapy. Cancer 115: 981-987.

10. Ross RW, Oh WK, Xie W, Pomerantz M, Nakabayashi M, et al. (2008) Inherited variation in the androgen pathway is associated with the efficacy of androgen-deprivation therapy in men with prostate cancer. J Clin Oncol 26: 842-847.
