# Supplementary material for: Genetic Variants in CASP3, BMP5, and IRS2 Genes May Influence Survival in Prostate Cancer Patients Receiving Androgen-Deprivation Therapy
Source: PLoS One. 2012 Jul 23;7(7):e41219. doi: 10.1371/journal.pone.0041219 (PMC3402522; doi:10.1371/journal.pone.0041219)
Supplement: Table S1 — Genotyped SNPs and the P values of their association with time to progression, PCSM, ACM during ADT. (DOC) [file pone.0041219.s002.doc]

**Table S1.** Genotyped SNPs and the *P* values of their association with time to progression, PCSM, ACM during ADT.

| SNP ID | Chromosome | Position | Associated Genes | Log-Rank *P* | | |
| --- | --- | --- | --- | --- | --- | --- |
|  |  |  |  | Progression | PCSM | ACM |
| rs12031994 | 1 | 240243350 | AKT3 | 0.199 | 0.360 | 0.534 |
| **rs3734444** | 6 | 55847512 | **BMP5** | **0.022** | **0.002** | **0.014** |
| **rs11597689** | 10 | 88639156 | **BMPR1A** | 0.174 | 0.252 | **0.028** |
| rs10906142 | 10 | 12478789 | CAMK1D | 0.235 | 0.292 | 0.173 |
| **rs4862396** | 4 | 185918821 | **CASP3** | 0.067 | 0.140 | **0.037** |
| rs2076003 | 1 | 11818413 | CLCN6|MTHFR | 0.076 | 0.482 | 0.869 |
| rs2075110 | 7 | 54993368 | EGFR | 0.087 | 0.288 | 0.412 |
| rs2371438 | 2 | 212791037 | ERBB4 | 0.249 | 0.243 | 0.194 |
| **rs2836370** | 21 | 38692234 | **ERG** | 0.869 | **0.035** | **0.019** |
| rs3936674 | 6 | 152259425 | ESR1 | 0.105 | 0.905 | 0.834 |
| rs10137185 | 14 | 63845529 | ESR2 | 0.660 | 0.939 | 0.520 |
| rs880774 | 3 | 193873452 | FGF12 | 0.302 | 0.665 | 0.214 |
| rs4775230 | 15 | 58091041 | FOXB1 | 0.287 | 0.105 | 0.407 |
| rs13241957 | 7 | 18581636 | HDAC9 | 0.155 | 0.812 | 0.192 |
| rs870549 | 1 | 24271302 | IL28RA | 0.088 | 0.155 | 0.163 |
| rs4147359 | 10 | 6148445 | IL2RA | 0.160 | 0.444 | 0.379 |
| rs12971499 | 19 | 7165282 | INSR | 0.663 | 0.196 | 0.079 |
| **rs7986346** | 13 | 109251608 | **IRS2** | **0.040** | **0.007** | **0.017** |
| **rs1972933** | 17 | 64952406 | **MAP2K6** | **0.043** | 0.727 | 0.448 |
| **rs10846667** | 12 | 123409304 | **NCOR2** | **0.011** | 0.452 | 0.172 |
| rs17024584 | 1 | 120223428 | NOTCH2 | 0.074 | 0.361 | 0.434 |
| rs1286762 | 3 | 25573913 | RARB | 0.645 | 0.880 | 0.437 |
| rs2253319 | 21 | 35109916 | RUNX1 | 0.052 | 0.586 | 0.569 |
| **rs3118536** | 9 | 134534407 | **RXRA** | 0.289 | **0.043** | **0.016** |
| rs2173049 | 1 | 2272168 | SKI | 0.953 | 0.770 | 0.696 |
| rs4625350 | 1 | 214931706 | TGFB2 | 0.529 | 0.575 | 0.520 |
| rs2107331 | 5 | 135405248 | TGFBI | 0.499 | 0.190 | 0.142 |
| rs10505346 | 8 | 120033024 | TNFRSF11B | 0.150 | 0.109 | 0.260 |
| rs3777018 | 5 | 82662675 | XRCC4 | 0.222 | 0.437 | 0.487 |
