# Supplementary material for: Genetic Variants in CASP3, BMP5, and IRS2 Genes May Influence Survival in Prostate Cancer Patients Receiving Androgen-Deprivation Therapy
Source: PLoS One. 2012 Jul 23;7(7):e41219. doi: 10.1371/journal.pone.0041219 (PMC3402522; doi:10.1371/journal.pone.0041219)
Supplement: Table S2 — Oligonucleotides used for genotyping analysis. (DOC) [file pone.0041219.s003.doc]

**Table S2.** Oligonucleotides used for genotyping analysis.

| SNP ID | Associated Genes | Well | Forward primer sequence | Reverse primer sequence | Extend primer sequence |
| --- | --- | --- | --- | --- | --- |
| rs12031994 | AKT3 | 1 | gTGTGTTTCATATACTGGTC | GTACATAATCTACTTCCAGGC | ggGGTCTTATTCTCTGGGTAGA |
| rs3734444 | BMP5 | 2 | TTTCCCGTCTTTCGTGGTTC | TGCAAAAGGAGGTTTGGGAG | CAATCATGTTCACTCCAG |
| rs11597689 | BMPR1A | 1 | GGCCAAAGCAAGATGCTTAG | GGTTCAAAACTTGTGACCAG | CTTTCCTTTATTTGTGCCT |
| rs10906142 | CAMK1D | 2 | GTTCAGCTTAGCACTTCCAG | AAGGTAGGGCTCTGACACAC | CCACGTAGGCTCCCC |
| rs4862396 | CASP3 | 1 | CTAGAGTATGAAGACCTGGC | ACAGGCCATAGTTCTCAAAC | GGCCATAGTTCTCAAACTTTAACAT |
| rs2076003 | CLCN6|MTHFR | 2 | GCTTGTGGAATTAGCTTTTG | CATTCTGATTCTTGGCCCAC | tctgTTGTCAAAATTAGCAAGCT |
| rs2075110 | EGFR | 2 | GTTTTCCCTCTGAAGACTCC | TTGCAAGACTGTCCTTCACC | CTGAAGACTCCAAAGAGTTA |
| rs2371438 | ERBB4 | 2 | GAGCAGGGAAGGTTTTAAAG | GGACTCAAGGCCTAGTTATC | ggggATGCTGCCAAAATTGTT |
| rs2836370 | ERG | 2 | CTTTACAGGTCAGATACCAA | TTCGCGACTCAGTTTGTACC | gcgggTCTCATAATCACAGTAGCAG |
| rs3936674 | ESR1 | 1 | TGGTGAAGAAGGAGGGAATG | ATAGAATTCCCCGCGCTCTG | CGCTCTGCCTTTCCTC |
| rs10137185 | ESR2 | 2 | AAGAGGAATAATACCTAGGC | CAACTGCACATTAAGCACTG | GTGTCATATGCTTTAGGTATGTTATTT |
| rs880774 | FGF12 | 1 | TCTAGGCAAGACACACTTGG | CAGCTGCACCTTTCTTATG | aCACCTTTCTTATGCATTTCT |
| rs4775230 | FOXB1 | 2 | TGAGATCTGATCTAAAGGAG | ACCTGGAAATAATACCAGAC | TAAAGGAGAATATATTTGGAGG |
| rs13241957 | HDAC9 | 1 | CATCAAACCCCACTCTTTAG | TGTATAACACTTTTGCACC | TCTTTAGAAATATAGATGATCCC |
| rs870549 | IL28RA | 2 | AAAGTGGTCCAGACTGCGAG | TTGGTCTTCTCTCTGCCTTG | TGCGAGACCGCGGAT |
| rs4147359 | IL2RA | 1 | AGCCACTGAAGCTGTCTAAC | TATGCCTCAATCTTCCCTGC | ccctCTCGGCCTCATCATCACAT |
| rs12971499 | INSR | 1 | AATGACCTCTTTCCTCCCTG | AATGTTTATGGAGGGCCAGC | ccctGGGCTCGGAACTTGTTTCTTCACA |
| rs7986346 | IRS2 | 1 | CAGTGTTAATTCTGTCCACC | CAATGACACCTTATTGGTGG | atTTTTCAATTTGTACACTTACTTT |
| rs1972933 | MAP2K6 | 1 | AGTCTCTCCATGTCTGCATC | CATTGCAGCTAAAGTGTGGG | ggCCTGGGCAACCACTG |
| rs10846667 | NCOR2 | 1 | ACTGCCACTCACATCATCTC | CAGTTTCCCCTTCTGTTGAG | tcCTCACATCATCTCATTTTTTTCTA |
| rs17024584 | NOTCH2 | 1 | CAACATTCCTGATTCAGGTG | GACTGGAAAATCCAGTTCCC | gtAAGCCTAGTAATATCTTCAGAGAC |
| rs1286762 | RARB | 2 | CCATGGATACCATTCCAATC | TTTCAGGCTCCAGAGAAGTG | aaggaTCCAGAGAAGTGATAGGCTG |
| rs2253319 | RUNX1 | 1 | GGTAGGTAAATCATGCACTC | TGAATCCTGACCCTGCTTTC | CCACATTTTGTAAGACATCTCG |
| rs3118536 | RXRA | 1 | ATTTCTGGCAGGGCCCAGT | AGGGACAGGCTCAGAGACAC | CAGAGACACCTGGGCAA |
| rs2173049 | SKI | 1 | ACCGTGTCTTGTAAAGGGTC | CAACTCTGGTAAAACAAGGC | gggcGGCTAAAAACAGCATTCTGC |
| rs4625350 | TGFB2 | 1 | TCTGTGGGACTGAAGTTTGC | TCTTGTACAGATGGATGAAG | TTGAACAGGAAGACTTTACAC |
| rs2107331 | TGFBI | 1 | AACCCTTACAGCTTTCTTTC | GGTTTAGGAGGAAGGGATAG | CTTGGAGTAGAGTAACTCA |
| rs10505346 | TNFRSF11B | 1 | TCTCTCTCTTGCTGTCTTCC | AAGTTATGCCATGCTTCCCC | ttccTCCCCTAGGGTGTCCTTTTA |
| rs3777018 | XRCC4 | 2 | GTCATCTCTGATGCTGTGTG | CAGCACAACAGAATCTACTTC | cACAGAATCTACTTCTATTGAGAACTT |
