# Supplementary material for: Genetic Variants in CASP3, BMP5, and IRS2 Genes May Influence Survival in Prostate Cancer Patients Receiving Androgen-Deprivation Therapy
Source: PLoS One. 2012 Jul 23;7(7):e41219. doi: 10.1371/journal.pone.0041219 (PMC3402522; doi:10.1371/journal.pone.0041219)
Supplement: Table S3 — Distribution of clinicopathologic characteristics and their associations with disease progression, PCSM, and ACM in prostate cancer patients receiving ADT. (DOC) [file pone.0041219.s004.doc]

**Table S3.** Distribution of clinicopathologic characteristics and their associations with disease progression, PCSM, and ACM in prostate cancer patients receiving ADT.

| Characteristic | Disease progression | | | PCSM | | | ACM | | |
| --- | --- | --- | --- | --- | --- | --- | --- | --- | --- |
|  | No, n (%) | Yes, n (%) | *P* | Alive, n (%) | Deceased, n (%) | *P* | Alive, n (%) | Deceased, n (%) | *P* |
| Total (n = 601) | 184 (30.7) | 415 (69.3) |  | 499 (83.2) | 101 (16.8) |  | 455 (75.8) | 145 (24.2) |  |
| Age at diagnosis, years | 73.3 | 71.9 | 0.078 | 72.3 | 72.6 | 0.748 | 71.8 | 74.0 | **0.008** |
| Clinical stage at diagnosis  T1/T2  T3/T4/N1/M1 | 70 (37.2)  113 (27.7) | 118 (62.8)  295 (72.3) | **0.019** | 176 (93.1)  320 (78.4) | 13 (6.9)  88 (21.6) | **<0.001** | 163 (86.2)  289 (70.8) | 26 (13.8)  119 (29.2) | **<0.001** |
| Gleason score at diagnosis  2-7  8-10 | 120 (32.2)  61 (28.5) | 253 (67.8)  153 (71.5) | 0.354 | 335 (89.6)  153 (71.5) | 39 (10.4)  61 (28.5) | **<0.001** | 308 (82.4)  137 (64.0) | 66 (17.6)  77 (36.0) | **<0.001** |
| PSA at ADT initiation, ng/mL |  |  |  |  |  |  |  |  |  |
| Median (IQR) | 35.0 (11.4-129) | | | | | | | | |
| <35  ≥35 | 100 (35.1)  81 (27.7) | 185 (64.9)  211 (72.3) | 0.057 | 262 (91.6)  216 (74.0) | 24 (8.4)  76 (26.0) | **<0.001** | 242 (84.6)  194 (66.4) | 44 (15.4)  98 (33.6) | **<0.001** |
| PSA nadir, ng/mL |  |  |  |  |  |  |  |  |  |
| Median (IQR) | 0.18 (0.01-1.33) | | | | | | | | |
| <0.2  ≥0.2 | 113 (37.7)  64 (21.9) | 187 (62.3)  228 (78.1) | **<0.001** | 281 (93.4)  212 (72.6) | 20 (6.6)  80 (27.4) | **<0.001** | 264 (87.7)  186 (63.7) | 37 (12.3)  106 (36.3) | **<0.001** |
| Time to PSA nadir, months |  |  |  |  |  |  |  |  |  |
| Median (IQR) | 10 (5-18) | | | | | | | | |
| <10  ≥10 | 72 (24.7)  105 (35.0) | 220 (75.3)  195 (65.0) | **0.006** | 228 (77.8)  265 (88.3) | 65 (22.2)  35 (11.7) | **0.001** | 204 (69.6)  246 (82.0) | 89 (30.4)  54 (18.0) | **<0.001** |

Abbreviations: PCSM, prostate cancer-specific mortality; ACM, all-cause mortality; ADT, androgen-deprivation therapy; PSA, prostate-specific antigen; IQR, interquartile range.

Subtotals do not sum to the total number of patients due to missing data.

*P* ≤ 0.05 are in boldface.
